# Beer‐spoilage characteristics of *Staphylococcus xylosus* newly isolated from craft beer and its potential to influence beer quality

**DOI:** 10.1002/fsn3.1256

**Published:** 2019-11-15

**Authors:** Zhimin Yu, Qiuying Luo, Li Xiao, Yumei Sun, Rong Li, Zhen Sun, Xianzhen Li

**Affiliations:** ^1^ School of Biological Engineering Dalian Polytechnic University Dalian China

**Keywords:** beer spoilage, craft beer, hop resistance, isolation, *Staphylococcus xylosus*

## Abstract

To meet demands for fresh flavor and unique taste from beer consumer, there is an increase in the popularity of craft beer, which is more susceptible to microbial contamination than the industry beer. A beer‐spoilage strain was isolated from craft beer and identified as *Staphylococcus xylosus* strain BS7. The isolate BS7 showed that high beer‐spoilage ability at low temperature (4°C), low pH (4.0) and high ethanol concentration (7.0%, v/v). Compared with the other known strains of *S. xylosus*, strain BS7 was resistant to hop compounds and had an evolutionary stability in hop resistance. Strain BS7 was able to grow quickly and utilizes nutrients in commercial beer, produces organic acids and biogenic amines, and changes beer flavor profile. These results suggest that *S. xylosus* strain BS7 is a beer‐spoilage strain with the danger, which can lead to the beer‐spoilage issues during craft beer production.

## Graphical abstract

*Staphylococcus xylosus* strain BS7 was isolated from turbid craft beer. Strain BS7 maintains the high beer‐spoilage ability at beer hurdle conditions and was resistant to hop compounds present in beer. Strain BS7 was able to produce organic acids, biogenic amines, and flavor compounds in beer.

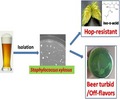

## 1 INTRODUCTION

Beer is an alcoholic beverage widely consumed by many people throughout the world (Yao, Zong, Cui, Mu, & Zhao, 2018). There is an increase in the popularity of craft beer, which is produced by microbreweries that are focused on the product with the fresh flavor and unique taste (Capece et al., 2018). Craft beer is a nonfiltered and unpasteurized product for maintaining the fresh sensory characteristic. So, compared to the industry beer, craft beer is more susceptible to microbial contamination that may cause the beer spoilage, such as turbidity, acidification, and bad flavor (Piacentini, Savi, Olivo, & Scussel, 2015).

Beer is considered as a microbiologically safe beverage because of a number of intrinsic antimicrobial hurdles, which include ethanol, hop bitter compounds, low pH, elevated carbon dioxide, low oxygen, and very little nutrients (Vriesekoop, Krahl, Hucker, & Menz, 2012). Nevertheless, some microorganisms still manage to grow in beer and cause beer turbidity and off‐flavor. This beer‐spoilage issue leads to economic loss to breweries and even a loss of consumer confidence (Manzano et al., 2011; Steiner, Becker, & Gastl, 2010). The beer‐spoilage bacteria include Gram‐positive lactic acid bacteria and Gram‐negative acetic acid bacteria, *Pectinatus* and *Megasphaera*, whose role and spoilage ability in the microbial system of beer was well researched (Suzuki, 2011). However, the occurrence and spoilage feature of the other contaminating microorganism in beer are not well documented. In some of the spoilage incidents, bacteria belong to genera *Staphylococcus*, *Bacillus*, *Enterobacter* and *Zymomonas* are potential beer‐spoilage bacteria, leading to the acidification, haze, sediment, ropiness, and off‐flavor of beer product (Munford et al., 2017; Suzuki, 2011). Therefore, research on the beer‐spoilage issue caused by beer‐spoilage bacteria is important for ensuring beer quality and brewery's economic interest.

*Staphylococcus xylosus* is a microorganism on the skin of humans or animals, and it is also a common bacterium naturally present in food or raw material (Dordet‐Frisoni, Dorchies, Araujo, Talon, & Leroy, 2007). Besides the ability of *S. xylosus* to form biofilm (Planchon et al., 2006), its ubiquity may be explained by the ability to adapt to various environmental conditions. *Staphylococcus xylosus* is naturally present in raw meat and milk and is used commonly as a starter culture for food fermentation (Blaiotta et al., 2004; Dordet‐Frisoni et al., 2007). The *Staphylococcus* species is defined as a nonpathogenic bacterium, but a few strains of *S. xylosus* are related to animal or human opportunistic infection (Akhaddar, Elouennass, Naama, & Boucetta, 2010; Wipf, Schwendener, & Perreten, 2014). Most bacteria adhered to skin or are used in food industry, while others could potentially be hazardous, showing the versatility of this species. A strain of *S. xylosus* was isolated from commercially turbid and off‐flavor craft beer, which had been recalled from local market by the breweries. This strain was able to grow well in the presence of hop compounds and displayed strong beer‐spoilage ability. To our knowledge, this was the first report of the beer‐spoilage performance of *S. xylosus*, although they had previously found in the homebrewed traditional beer of South Africa (Lues et al., 2011).

Therefore, investigating the effects of beer environmental conditions on the beer‐spoilage ability of *S. xylosus* and its influences on beer quality would be helpful to better understand the beer‐spoilage feature by contaminating bacteria and allowing the prevention from beer microbiological instability by spoilage bacteria and to ensure good beer quality.

## 2 MATERIALS AND METHODS

### 2.1 Strains, materials, and culture conditions

*Staphylococcus xylosus* AS1.8382 was used as a reference strain and obtained from the China General Microbiological Culture Collection Centre (CGMCC). *Staphylococcus xylosus* AS1.8382 was incubated on MRS agar plate at 30°C.

Ten bottles of craft beer were obtained from the Gins Breweries Co. Ltd., which had been recalled from the Chinese market in 2017 because of turbidity and off‐flavor. The craft beer was nonpasteurized and non‐sterile‐filtered product, and was pilsner‐type beer (330 ml, 4.3% v/v ethanol, pH 4.2, 18 bitterness units). Commercial bottled beer (pasteurization, 500 ml, 3.6% v/v ethanol, pH 4.1, 15 bitterness units) was pilsner‐type product and was purchased from local supermarket in Dalian, China.

Isomerized hop extract was purchased from Hopsteiner Trading (Zhuhai) Co. Ltd. and was used for hop compound preparation. The iso‐*α*‐acid content is 30.8% (w/w) in hop extract. The concentration of hop compounds in medium is expressed as the concentration of iso‐*α*‐acids.

Advanced beer detection (ABD) agar plate (per litter) was composed of 10.0 g glucose, 5.0 g peptone, 4.0 g meat extract, 2.0 g yeast extract, 1.0 g diammonium hydrogen citrate, 1.0 g sodium acetate, 1.0 g dipotassium hydrogen phosphate, 0.5 g polysorbate 80, 0.1 g magnesium sulfate, 0.02 g manganese sulfate, 0.1 g cycloheximide, 15.0 g agar, and 1,000 ml commercial beer at pH 5.0.

### 2.2 Isolation and identification of beer‐spoilage strains

Each bottle of beer (330 ml) was membrane‐filtered (cellulose nitrate, *Φ* 50 mm × 0.45 μm, Jinteng) using vacuum filtration. The membranes were placed onto ABD agar plate. These plates were incubated at 30°C under anaerobic condition (Anaerobic Workstation, Whitley A35; Don Whitley Scientific Co. Ltd.) and were continuously observed for 2 weeks until the colonies were large enough for picking. After incubation, each colony on ABD plate was transferred to the slant of MRS agar and then incubated at 30°C under anaerobic condition. These slants were stored at 4°C. The isolates were inoculated into commercial beer. The inoculated beer was incubated at 30°C and examined regularly for growth property during 2 months. Beer sample without inoculation was used as control.

Each isolate was examined for cell shape, size, motility, and Gram staining with a phase‐contrast microscope (Leica Microsystems) and transmission electron microscope (JEOL Ltd.). Three percent hydrogen peroxide was used to test for catalase activity. The production of acid and gas from carbohydrate was determined in the basal medium supplemented with various carbohydrates, as previously described (Smibert & Krieg, 1994). Carbohydrate utilization was determined as follows: Cells grown in MRS medium were collected by centrifugation at 8,000 *g* for 10 min. The cells were then washed and resuspended in sterile deionized water. They were inoculated into API Staph medium (BioMerieux, France) at a concentration of 1.0 × 10^8^ cells/ml, and poured into the wells of an API Staph plate. The color change of each well was observed for up to 10 days.

The 16S rRNA gene sequences and phylogeny of isolates were analyzed according to the method as described by Wang, Liu, Sun, Du, and Li (2017).

### 2.3 Effects of environmental conditions on beer‐spoilage ability of isolate BS7

Isolate BS7 was activated by culturing in MRS broth at 30°C under anaerobic condition for 3 days (growth logarithmic phase), and then, the culture fluid was diluted to an OD_600_ of 0.2 with commercial beer. The cell suspension was inoculated at 0.1% (v/v) inoculum in commercial beer and was incubated at 4, 10, 20, 30, or 37°C for 7 days. The culture beer was sampled and determined turbidity by spectrophotometry at OD_600_ to evaluate beer‐spoilage ability. Effect of pH value on beer‐spoilage ability was determined after culturing isolate in commercial beer with pH 3.0, 4.0, 5.0, 6.0, or 7.0 at 30°C for 7 days. The pH value of commercial beer was adjusted with 0.1 M hydrochloric acid or 0.1 M sodium hydroxide. Effect of ethanol content on beer‐spoilage ability was determined after culturing isolate BS7 in commercial beer with different concentrations of ethanol (4.0, 5.0, 6.0, 7.0, 8.0, or 9.0%, v/v) at 30°C for 7 days. Beer sample without inoculation was used as control (OD_600_ = 0.06).

### 2.4 Minimum inhibitory concentration (MIC) and hop‐resistant ability of isolate BS7

The culture fluid isolate BS7 was diluted to an OD_600_ of 0.2 with sterile deionized water. A 50 μl of the cell suspension was spotted on MRS plate, containing 0–800 μM of iso‐*α*‐acids of hop compounds. After 7 days of incubation at 30°C, all colonies were enumerated on the agar plate. The concentration of iso‐*α*‐acids of the MRS plate nongrowing colony was defined as MIC of iso‐*α*‐acids for BS7. The hop‐sensitive *S. xylosus* AS1.8382 was cultured on MRS plate containing iso‐*α*‐acids of hop compounds as the reference.

Isolate BS7 was successive transplantation for more than 20 generations in MRS broth nonhop compounds. The cell suspension of subcultured strain and original strain (OD_600_ = 0.2) was inoculated at 0.1% (v/v) inoculum in commercial beer added with the hop compounds at 0, 100, 200, 300, 400, 500, or 600 μM as the concentration of iso‐*α*‐acids at 30°C for 7 days. The culture beer was sampled, and its turbidity was determined using spectrophotometry at OD_600_ to evaluate hop‐resistant ability. Beer sample without inoculation was used as control (OD_600_ = 0.06).

### 2.5 Analysis fermentation performance of isolate BS7

The cell suspension of isolate BS7 (OD_600_ = 0.2) was inoculated at 0.1% (v/v) inoculum in commercial beer and was incubated at 30°C for 7 days. The cultured beer was sampled every day and then spread on MRS agar plate for colony counting assay. The cultured beer was ultrasonicated (Ultrasonic Cleaner DS8510DT, Shengxi) for 20 min removing CO_2_ in beer and centrifuged at 8,000 *g* for 15 min. The supernatant every day was used to analyze the pH value, free amino nitrogen (FAN) content, and residual sugar content. The supernatant at end of fermentation was used to analyze the concentration of organic acids, biogenic amines, and flavor compounds. The uninoculated commercial beer was incubated at 30°C as the control group.

### 2.6 Analysis of free amino nitrogen and residual sugars

The FAN content for all samples was analyzed by a ninhydrin‐based method (Lan et al., 2016). Absorbance was measured at OD 570 nm. Glycine was used as a standard.

The residual sugar content for all samples was analyzed by a 3,5‐dinitrosalicylic acid colorimetric method (Lei, Zhao, Yu, & Zhao, 2012). Absorbance was measured at OD 540 nm. Glucose was used as a standard.

### 2.7 Analysis of organic acids, biogenic amines, and flavor compounds

The cultured beer samples were filtered using a 0.22‐μm membrane, and then, the concentration of organic acid was analyzed by HPLC method using an Agilent 1260 Infinity HPLC system (Agilent Technologies), a UV detector (Agilent Technologies), and a nonpolar C18 column (4.6 × 250 mm; Agilent Technologies) (Wang et al., 2017). The mobile phase was composed of 0.025 M (NH_4_)_2_HPO_4_–H_3_PO_4_buffer (pH 2.5) and acetonitrile (95:5) and used at 30°C at a flow rate of 1.0 ml/min. The injection volume was 20 μl, and the determination wavelength was 214 nm.

The concentration of biogenic amines was detected as described by Lorencová et al. (2012). A 10 ml beer sample and 3 g NaCl were mixed and adjusted to pH 12 with 3 M NaOH. A 1 ml sample and 1 ml normal butanol and chloroform solution (1:1, v/v) were mixed and vortexed for 10 min. The upper layer was collected, and the lower layer was extracted again after centrifugation at 8,000 *g* for 15 min at 4°C. The extract was acidified using 2 ml of 1 M HCl and then dried by N_2_ at 45°C. The dried sample was dissolved in 1 ml of 0.2 M HCl and added 1.5 ml saturated NaHCO_3_ and 1 ml dansyl chloride, and it reacted at 60°C for 40 min. A 100 μl sodium glutamate was added to the reaction solution, and the mixture and 1 ml pure water were mixed after incubation for 15 min, and dried to 3 ml using N_2_ at 45°C. The reaction solution was extracted using 3 ml ether, and the upper layer was collected. The extract was dried by N_2_ at 45°C. The dried extract was dissolved in 1 ml methanol, and the solution was filtered using 0.22‐μm membrane filter. A 20 μl samples were injected into the HPLC system (Agilent Technologies) for the detection of biogenic amines. A nonpolar C18 column (4.6 × 250 mm; Agilent Technologies) was used at 30°C at a flow rate of 0.8 ml/min. The mobile phase consisted of pure water and methanol (8:2, v/v). The determination wavelength was 255 nm.

A 5 g of beer sample was added into 15‐ml glass vial containing 2 g of NaCl. These glass vials were subsequently sealed with PTFE‐silicone septa (Supelco).

Headspace solid‐phase microextraction with an 80‐μm carboxen–polydimethylsiloxane fiber was used. The sample vials were equilibrated for 15 min at 45°C followed by fiber exposure to the headspace for 25 min. The analysis was performed with trace gas chromatography–mass spectrometry system consisted of an Ultra GC (Thermo Finnigan), a TR‐5MS column (30 m × 0.25 mm × 0.25 μm film thickness, J&W Scientific), and a quadrupole DSQ II MS. Helium was used as a carrier gas with the flow rate of 0.8 ml/min. The oven temperature program was 8 min at 45°C and then 2°C/min to 200°C (Zhao et al., 2016).

### 2.8 Statistical analysis

All tests were performed in triplicate. Data are expressed as the mean ± *SD*. Analysis of variance and significant differences among means were tested by independent‐sample *t* test (*p* < .05) using SPSS software (version 17.0 for Windows, SPSS Inc).

## 3 RESULTS AND DISCUSSION

### 3.1 Isolation and identification of beer‐spoilage bacteria

Forty‐five colonies were isolated from the ABD plates and were inoculated into commercial beer; of these, 38 isolates could cause beer turbidity and were named as BS1–38. An NCBI BLAST comparison for 16S rRNA sequence revealed that isolate BS7 matched best with the genus *Staphylococcus*, the percentage identity was 99%, and that other 37 isolates matched best with the genus *Lactobacillus,* the percentage identity was 99%. As *Staphylococcus* strain is uncommon beer‐spoilage bacteria (Suzuki, 2011), we selected this *Staphylococcus* isolate for further study.

The 16S rRNA gene sequence from isolate BS7 was identical. When compared with the available sequences in the GenBank/EMBL database, the sequence of BS7 was 99% homologous to the 16S rRNA from type strain *S. xylosus*.

Strain BS7 was coccoid, nonmotile cells, with diameters of 0.5–1 µm. BS7 was Gram‐positive, facultatively anaerobic, and catalase‐negative. Acid was produced from glucose and maltose, but not from mannose and galactose. BS7 could not produce gas from glucose. The carbohydrate utilization patterns of BS7 were consistent with the key characteristics of *S. xylosus*.

### 3.2 Effects of temperature, pH, and ethanol content on beer‐spoilage ability of isolate BS7

Beer is intrinsically resistant against growth of spoilage and pathogenic microorganisms because of a number of inhibitory factors, including temperature, pH, and ethanol content (Vriesekoop et al., 2012). The beer‐spoilage ability of isolate BS7 in commercial beer was determined after 7 days of incubation at different temperatures, pH values, and ethanol contents.

**Figure 1:**
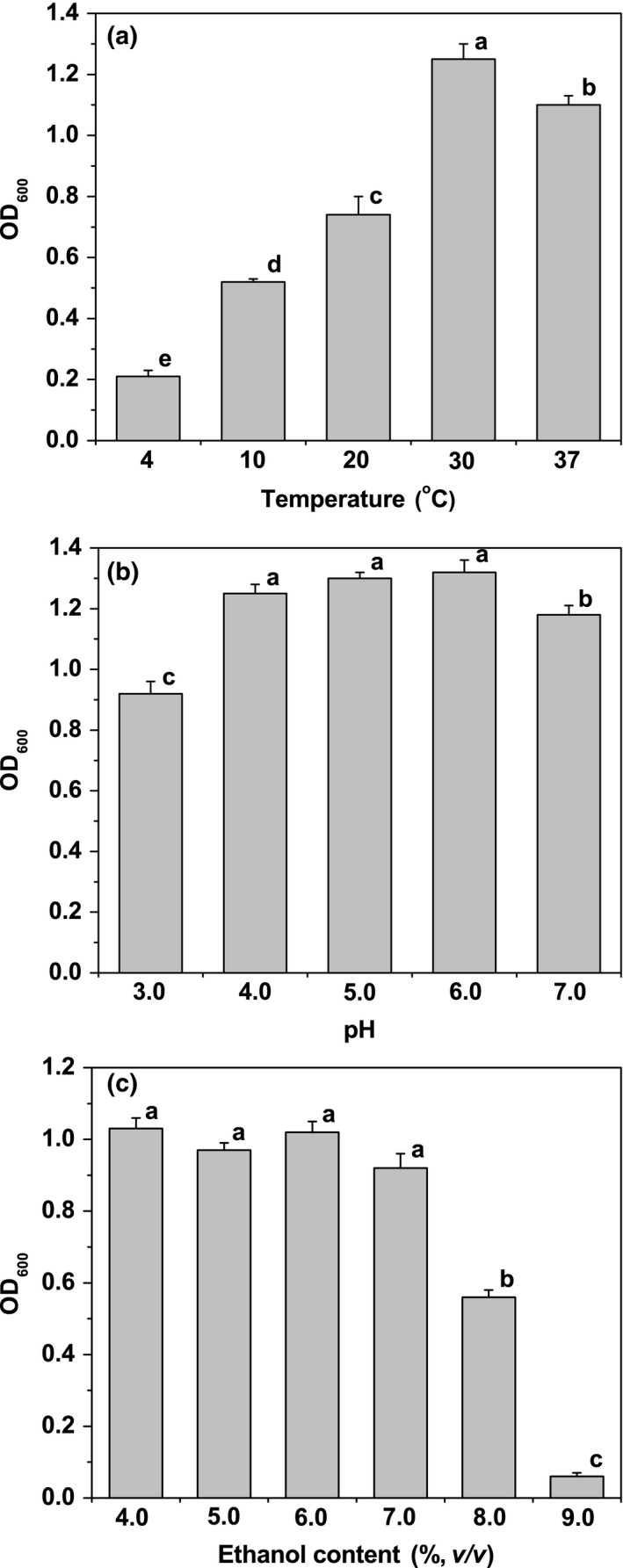
The beer‐spoilage ability of *Staphylococcus xylosus* strain BS7 in commercial beer at different temperatures (a), at different pH values (b), and at different ethanol contents (c). Each number represents the mean ± *SD* of three replicates. Locations for each column marked by different letters are significantly different (*p* < .05)

As shown in Figure 1a, the beer turbidity of isolate BS7 at 4–37°C was higher than that at the beginning of fermentation (OD_600_ = 0.067), indicating that isolate BS7 had a potential for contamination and proliferation in beer at different storage temperatures. Temperature is an important factor in the determination of cell growth in beer (Menz, Aldren, & Vriesekoop, 2011). The beer‐spoilage ability of isolate BS7 decreased significantly (*p* < .05) with decreasing temperature from 30°C to 4°C, which indicated that low temperature helped to protect beer from spoilage by *S. xylosus*.

Most beer has a relatively low pH (range 3.4–4.8) (Edgerton, 2005). Low pH value in beer damages cell enzyme system and also enhances the inhibitory effect of hops (Beales, 2004). As shown in Figure 1b, isolate BS7 could grow in commercial beer with initial pH 3.0–7.0. The optimum pH for growth of isolate BS7 was pH 4.0–6.0, while lower turbidity was detected at pH 3.0 than at the other pH values. Compared with other known strains of *S. xylosus* growing at above pH 5.0 (Dordet‐Frisoni et al., 2007), isolate BS7 could grow at lower pH.

The conversion of carbohydrate to ethanol (0.5%–10%, v/v) by yeast cell during the wort fermentation provides one of the major antimicrobial hurdles (Menz et al., 2011). As shown in Figure 1c, there was no significant difference (*p* > .05) in the beer‐spoilage ability when the final concentration of ethanol was less than 7.0% (v/v) in commercial beer. Nevertheless, a significant decrease (*p* < .05) in the beer turbidity was observed when the ethanol content was above 7.0% (v/v). In general, the ethanol content in beer is not more than 7.0% (v/v). Therefore, it can be concluded that ethanol in beer does not affect beer spoilage caused by isolate BS7.

### 3.3 Hop‐resistant ability of isolate BS7

Beer contains bitter hop compounds (approximately 17–55 mg/L of iso‐*α*‐acids) that are toxic, especially against Gram‐positive bacteria (Yansanjav et al., 2004). Hop‐resistant ability is considered to be caused by acquired immunity after prolonged contact with hop compounds under brewing condition (Vriesekoop et al., 2012). It is a necessity for the beer‐spoilage bacteria to acclimatize to beer or hop compounds in order to reproduce in beer (Suzuki, Sami, Iijima, Ozaki, & Yamashita, 2006). The MIC of iso‐*α*‐acids for isolate BS7 was 600 μM at 30°C after 7 days of incubation. In contrast, the reference strain of *S. xylosus* AS1.8382 derived from fermented fish could not grow on MRS plate containing iso‐*α*‐acids at 50 μM, indicating that this strain was sensitive to hop compounds. This result showed that isolate BS7 was able to survive and grow in a wide range of hop compound concentration, indicating that this isolate was hop‐resistant.

**Figure 2:**
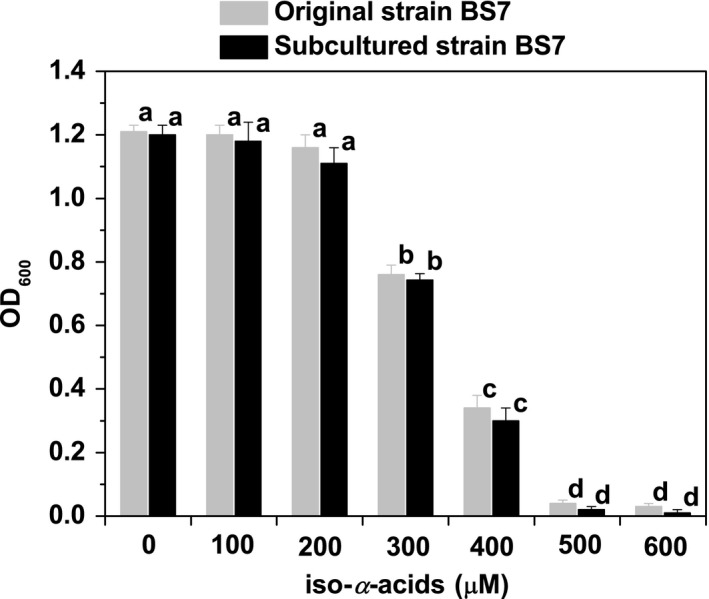
The hop‐resistant ability of the original strain *Staphylococcus xylosus* BS7 and subcultured strain *S. xylosus* BS7 in commercial beer supplemented with hop compounds in the range of 0–600 μM as the concentration of iso‐*α*‐acids at 30°C for 7 days. Each number represents the mean ± *SD* of three replicates. Locations for each column marked by different letters are significantly different (*p* < .05)

Strain BS7 was serially subcultured 20 times on MRS agar medium without hop compounds, following which the cell growth was determined in the commercial beer supplemented with hop compounds. As shown in Figure 2, subcultured strain BS7 could grow well in the commercial beer supplemented with hop compounds as the concentration of iso‐*α*‐acids in the range of 0–200 μM. The biomass of subcultured strain BS7 decreased with increasing iso‐*α*‐acid concentration above 200 μM. Compared to control sample (beer without inoculation), the OD_600_ value of subcultured strain BS7 (0.065) was not significantly different. This result indicated that this strain does not grow when the concentration of iso‐*α*‐acids reached 500 μM. Furthermore, subcultured strain BS7 in beer supplemented with hop compounds showed no significant difference (*p* > .05) in the cell growth to that of original strain BS7, indicating that strain BS7 is stable in terms of hop resistance. This result is different from that of *Lactobacillus* strains, the hop resistance of which decreased upon prolonged serial subculture in the absence of hop compounds (Sakamoto & Konings, 2003). However, there was no attenuation in the hop resistance of isolate BS7, indicating that hop resistance can be a very stable property. Fernandez and Simpson (1993) reported that the hop resistance in *Lactobacillus brevis* strain BSO310 could not be altered by plasmid curing or mutation induced with ultraviolet light, suggesting that it may be generally a stable character, both phenotypically and genetically. In addition, the result showed that the inhibition concentration of iso‐*α*‐acids against BS7 in beer (500 μM) was lower than MIC of iso‐*α*‐acids for BS7 on MRS plate (600 μM), which was caused by the low pH (4.1) in beer that could enhance the inhibitory effect of hops against spoilage bacteria (Beales, 2004).

### 3.4 Fermentation performances in commercial beer by isolate BS7

**Figure 3:**
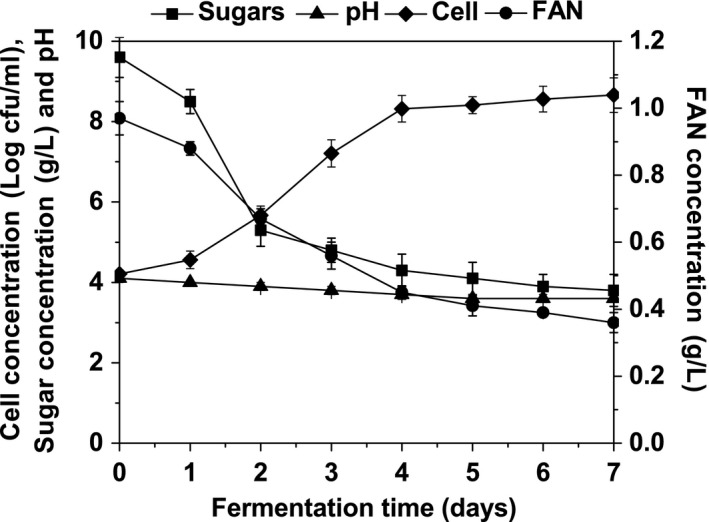
Fermentation profiles of *Staphylococcus xylosus* strain BS7 in commercial beer at 30°C for 7 days. Each number represents the mean ± *SD* of three replicates

The process of beer fermentation by isolate BS7 was evaluated by utilization of carbon and nitrogen source, production of acid metabolites, and cell growth. As shown in Figure 3, isolate BS7 could grow in beer and reach the maximum cell concentration within 4 days, showing it had strong beer‐spoilage ability. The residual sugars and free amino nitrogen (FAN) in commercial beer were utilized by isolate BS7 during beer fermentation (Figure 3). The concentrations of nutritive substances available for the growth of contaminating microorganisms, such as carbohydrate, amino acids, and some B‐vitamins, are very low in commercial beer, which is the least prone for beer spoilage (Vriesekoop et al., 2012). However, isolate BS7 showed good growth performance and the utilization of nutrient in commercial beer. Isolate BS7 can resistant several antimicrobial hurdles in commercial beer, such as ethanol, hop bittering compounds, and low pH, indicating that it can preserve the transmembrane pH gradient and prevent the intracellular acidification required for the uptake of nutrients (Sakamoto & Konings, 2003). As shown in Figure 3, the pH value in commercial beer incubated with isolate BS7 went down from 4.1 to 3.6 during beer fermentation, which was due to the fact that isolate BS7 formed acid metabolites during beer fermentation.

### 3.5 Changes in organic acids, biogenic amines, and flavor compounds in commercial beer incubated with isolate BS7

Contaminating microorganisms spoil beer by producing haze or rope and causing off‐flavor issues, such as sourness and atypical odor (Sakamoto & Konings, 2003). Therefore, the changes in characteristic components in commercial beer were detected when isolate BS7 was cultured for 7 days. As shown in Table 1, the content of total organic acids in inoculated beer was significantly higher (*p* < .05) than that of control group (beer incubated without strain BS7). The content of two organic acids, including lactic acid and succinic acid, increased markedly (*p* < .05) in inoculated beer. However, the content of four organic acids, including citric acid, malic acid, oxalacetic acid, and pyruvic acid, decreased markedly (*p* < .05) in inoculated beer. There was no significant difference (*p* > .05) in the content of the other organic acids, including α‐ketoglutaric acid and glyoxylic acid, between inoculated beer and control group (Table 1). This result indicated that isolate BS7 can utilize and form organic acids in commercial beer. As discussed earlier, the metabolism of organic acids in beer‐spoilage bacteria is known to directly or indirectly enhance energy production and proton motive force (PMF) generation in commercial beer where nutrient is scarce. Increased organic acids can result in the low pH and bad taste in beer, and impact severely the beer flavor profiles and forming ability (Wang et al., 2017).

**Table 1:** Changes in flavor volatiles, organic acids, and biogenic amines in bottled commercial beer following incubation with *Staphylococcus xylosus* strain BS7 at 30°C for 7 days

Components (mg/L)	Control	Strain BS7
Organic acids		
Citric acid	123.37 ± 3.01	110.19 ± 3.89*
Lactic acid	61.44 ± 0.89	124.77 ± 2.22*
Succinic acid	52.38 ± 1.12	112.31 ± 2.54*
Malic acid	124.70 ± 2.56	99.20 ± 1.87*
Oxalacetic acid	2.48 ± 0.04	1.82 ± 0.03*
α‐Ketoglutaric acid	84.27 ± 1.53	86.57 ± 1.75
Glyoxylic acid	24.51 ± 0.84	23.19 ± 0.57
Pyruvic acid	43.53 ± 1.10	6.33 ± 0.21*
Total organic acids	516.68 ± 7.69	564.38 ± 9.36*
Biogenic amines		
Tryptamine	7.68 ± 0.11	8.83 ± 0.26*
Putrescine	2.17 ± 0.04	2.16 ± 0.03
Cadaverine	0.67 ± 0.01	0.83 ± 0.02*
Histamine	0.14 ± 0.01	0.12 ± 0.01
Tyramine	0.71 ± 0.03	0.74 ± 0.02
Spermidine	0.34 ± 0.01	0.35 ± 0.01
Spermine	0.03 ± 0.00	0.04 ± 0.01
Total biogenic amines	11.73 ± 0.25	13.09 ± 0.32*
Flavor volatiles		
Ethyl formate	0.53 ± 0.01	0.51 ± 0.02
Ethyl acetate	17.49 ± 0.38	17.74 ± 0.45
Isobutyl acetate	0.59 ± 0.02	0.60 ± 0.03
Isoamyl acetate	0.38 ± 0.02	0.41 ± 0.01
Ethyl caprylate	0.73 ± 0.03	0.69 ± 0.02
Total esters	19.75 ± 0.62	19.93 ± 0.70
Isobutanol	8.81 ± 0.16	9.33 ± 0.20*
*n*‐Propanol	0.09 ± 0.00	0.09 ± 0.00
Butanol	0.22 ± 0.01	0.23 ± 0.01
Isopentanol	43.47 ± 0.95	47.25 ± 1.36*
2,3‐Butanediol	6.57 ± 0.23	6.90 ± 0.13
Total alcohols	59.16 ± 1.20	63.80 ± 0.99*

Note

Each number represents the mean ± *SD* of three replicates.

* Significant difference between control and strain BS7 at *p* < .05.

Beer represents a fermented product with potentially higher concentration of biogenic amines, which might threaten human health (Loret, Deloyer, & Dandrifosse, 2005). Biogenic amine is mainly formed as a result of decarboxylase activity of contaminating bacteria in beer originating probably in poor hygiene of manufacturing facilities or in contaminated ingredients (Kalač, Savel, Křížek, Pelikánová, & Prokopová, 2002; Spano et al., 2010). Seven biogenic amines were identified by derivatization method in the beer incubated with isolate BS7. Compared to the control group, the level of tryptamine and cadaverine in the beer incubated with isolate BS7 was significantly (*p* < .05) increased. The level of the other biogenic amines was not remarkably (*p* > .05) changed (Table 1). This result indicated that isolate BS7 was a producer of biogenic amines during beer fermentation. Previous researches (Gücükoğlu & Küplülü, 2010; Kung et al., 2016; Martuscelli, Crudele, Gardini, & Suzzi, 2000) have also revealed that the genus *Staphylococcus* isolated from fermented food can produce several biogenic amines, such as histamine, tyramine, putrescine, cadaverine, tryptamine, phenylethylamine, spermine, and/or spermidine.

Generally, beer‐spoilage bacteria have the ability to produce a variety of undesirable flavor and aroma, and high turbidity to final products (Wang et al., 2017). Compared to the control group, the content of ester volatiles in the beer incubated with isolate BS7 was not remarkably changed (*p* > .05) (Table 1). As shown in Table 1, isolate BS7 resulted in significant increases (*p* < .05) in the concentration of isobutanol and isopentanol compared to the control group, leading to unbalanced beer flavor. The other detected alcohol volatiles were not significantly affected (*p* > .05) by the presence of isolate BS7. Similar to *L. brevis*, *S. xylosus* strain BS7 could also synthesize some flavor compounds in beer (Vriesekoop et al., 2012), such as causing an increase in higher alcohols. Isobutanol and isopentanol in beer contribute to stimulating alcohol and bitter almond flavor and are a key reason headache after beer drinking (Kreisz, 2009). So, the effects of higher alcohols on flavor of beer were most often negatively accepted by consumer when its content was excessive (Yu et al., 2012).

## 4 CONCLUSION

*Staphylococcus xylosus* BS7 was isolated from craft beer. This stain showed strong growth in beer supplemented with different hop concentrations and possessed beer‐spoilage characteristics, such as rendering beer turbid and producing organic acids and biogenic amines. It could also synthesize produced flavor substances in beer, resulting in changes in beer flavor. Therefore, we conclude that *S. xylosus* BS7 is a beer‐spoilage strain with strong beer‐spoilage ability.

## CONFLICT OF INTEREST

We declare that we have no conflict of interest.

## ETHICAL APPROVAL

This study does not involve any human or animal testing.
